# Prevalence of Hypertension and Its Associated Risk Factors among 34,111 HAART Naïve HIV-Infected Adults in Dar es Salaam, Tanzania

**DOI:** 10.1155/2016/5958382

**Published:** 2016-10-30

**Authors:** Marina Njelekela, Alfa Muhihi, Akum Aveika, Donna Spiegelman, Claudia Hawkins, Catharina Armstrong, Enju Liu, James Okuma, Guerino Chalamila, Sylvia Kaaya, Ferdinand Mugusi, Wafaie Fawzi

**Affiliations:** ^1^Department of Physiology, Muhimbili University of Health and Allied Sciences, Dar es Salaam, Tanzania; ^2^Management and Development for Health, HIV/AIDS Care and Treatment Program, Dar es Salaam, Tanzania; ^3^Africa Academy for Public Health, P.O. Box 79810, Dar es Salaam, Tanzania; ^4^Department of Epidemiology, Harvard TH Chan School of Public Health, Boston, MA, USA; ^5^Department of Biostatistics, Harvard TH Chan School of Public Health, Boston, MA, USA; ^6^Feinberg School of Medicine, Northwestern University, Chicago, IL, USA; ^7^Tufts University School of Medicine, Boston, MA, USA; ^8^Department of Nutrition, Harvard TH Chan School of Public Health, Boston, MA, USA; ^9^Department of Global Health and Population, Harvard TH Chan School of Public Health, Boston, MA, USA; ^10^Department of Psychiatry and Mental Health, Muhimbili University of Health and Allied Sciences, Dar es Salaam, Tanzania; ^11^Department of Internal Medicine, Muhimbili University of Health and Allied Sciences, Dar es Salaam, Tanzania

## Abstract

*Background*. Elevated blood pressure has been reported among treatment naïve HIV-infected patients. We investigated prevalence of hypertension and its associated risk factors in a HAART naïve HIV-infected population in Dar es Salaam, Tanzania.* Methods*. A cross-sectional analysis was conducted among HAART naïve HIV-infected patients. Hypertension was defined as systolic blood pressure (SBP) ≥ 140 mmHg and/or diastolic blood pressure (DBP) ≥ 90 mmHg. Overweight and obesity were defined as body mass index (BMI) between 25.0–29.9 kg/m^2^ and ≥30 kg/m^2^, respectively. We used relative risks to examine factors associated with hypertension.* Results*. Prevalence of hypertension was found to be 12.5%. After adjusting for possible confounders, risk of hypertension was 10% more in male than female patients. Patients aged ≥50 years had more than 2-fold increased risk for hypertension compared to 30–39-years-old patients. Overweight and obesity were associated with 51% and 94% increased risk for hypertension compared to normal weight patients. Low CD4+ T-cell count, advanced WHO clinical disease stage, and history of TB were associated with 10%, 42%, and 14% decreased risk for hypertension.* Conclusions*. Older age, male gender, and overweight/obesity were associated with hypertension. Immune suppression and history of TB were associated with lower risk for hypertension. HIV treatment programs should screen and manage hypertension even in HAART naïve individuals.

## 1. Introduction

The introduction of Highly Active Antiretroviral Therapy (HAART) marked a milestone in the prognosis, course of infection, and quality of life for people living with human immunodeficiency virus (HIV) in both developed and developing countries [1, 2]. Cardiovascular diseases (CVDs) are increasingly observed in HIV-infected people especially in developed countries where people with HIV/AIDS are living much longer [3]. In addition to increased patient longevity, data indicate that increased incidence of CVDs is also attributable to HIV infection itself and use of HAART [4, 5].

Generally, the impact of HIV and HAART on hypertension remains controversial. A recent systematic review with meta-analysis of over 44,000 HIV-infected patients has shown the mean systolic blood pressure and risk of hypertension to be significantly higher among HAART exposed compared to HAART naïve individuals [6]. Studies conducted in Europe and US found higher rates of hypertension among HIV-infected adults on HAART than uninfected adults [7] with others indicating no difference [8, 9]. However, results from a multicenter AIDS cohort study of men with blood pressure measurements between 1984 and 2003 indicated a significant association between prolonged use of HAART with systolic hypertension [10].

A systematic review and meta-analysis from sub-Saharan Africa found lower blood pressure levels among HIV-infected than uninfected adults [11]. Another large, population-based study from South Africa indicated hypertension to be less common among HIV-infected adults [12]. However, both studies from Africa lacked data on long-term HAART use. Hypertension may develop as a result of long-term HAART use due to weight gain, drug toxicity, and some immune-related phenomenon. A study conducted in Nigeria reported no association between HIV infection and HAART status with hypertension [13]. However, this study had a relatively small sample size and was of short duration of HAART in the treated group. Reports from other studies indicate a slightly higher prevalence of CVD risk factors among HIV-infected individuals, especially those on HAART [14, 15].

Data from resource-limited settings among non-HIV-infected people has indicated increasing rates of traditional risk factors associated with CVD such as hypertension, diabetes mellitus, and dyslipidemia, primarily as a result of obesity and urbanization [16–18]. These risk factors contribute to a significant proportion of overall disease burden in Africa [19, 20]. The prevalences of metabolic syndrome and hypertension are reported to be increasing in Africa [18, 21]. Metabolic syndrome is defined as a cluster of several cardiometabolic risk factors, including abdominal obesity, hyperglycemia, dyslipidemia, and elevated blood pressure [22].

In view of existing gap of knowledge and scarcity of data on the prevalence and correlates of CVD risk factors especially among HIV-infected population in resource-limited settings, we conducted this analysis to assess the prevalence of hypertension and its associated risk factors in a cohort of HIV-infected, HAART naïve in Dar es Salaam, Tanzania.

## 2. Methods

### 2.1. Study Design, Site, and Population

Data for this cross-sectional analysis were collected at 12 HIV Care and Treatment Clinics (CTCs) affiliated to Management and Development for Health (MDH) and supported by President's Emergency Plan for AIDS Relief (PEPFAR) in Dar es Salaam, Tanzania. The MDH-PEPFAR program was established in 2004 and provided infrastructure, laboratory, and technical support to HIV CTCs, Prevention of Mother to Child Transmission (PMTCT) clinics, and Tuberculosis facilities in Dar es Salaam region. Dar es Salaam has a population of more than four million people with an HIV prevalence of 6.9% [23]. This analysis is comprised of patients who had blood pressure (BP) measurement taken, were ≥15 years of age, and were nonpregnant at the time of enrolment.

### 2.2. Blood Pressure Measurement

Blood pressure measurements were taken on arrival to the clinic using a standardized digital blood pressure measuring machine (AD Medical Inc.). Three blood pressure readings were taken on the left upper arm with the participant in a seated position following at least 5 to 10 minutes of rest. The average of the three readings was used in this analysis.

### 2.3. Anthropometric Measurement

Body weight and height were taken following standard procedures. Body weight (to the nearest 0.5 kg) was taken with the participant in light clothing using a SECA scale. Height (to the nearest 0.5 cm) was measured using a stadiometer with participants wearing no shoes. Body mass index (BMI) was then calculated as weight in kilograms divided by square of height in meters (kg/m^2^). Overweight was defined as BMI between 25.0 and 29.9 kg/m^2^ and obesity as BMI ≥ 30 kg/m^2^.

### 2.4. Clinical and Laboratory Procedures

Clinical care of all HIV-infected patients at MDH-supported CTCs follows the Tanzanian National guidelines for management of HIV patients [24]. Following HIV diagnosis, patients were enrolled in the CTC clinic and had WHO clinical stage, CD4+ T-cell count, and HAART eligibility status determined. Eligible patients were subsequently initiated on HAART after adherence counseling and were followed up by physicians and nurses at two weeks after HAART initiation for assessment and management of any toxicity. They were then assessed at monthly clinic visits. Patients not yet meeting HAART initiation criteria were followed up at 6 monthly HIV care and monitoring visits.

Blood samples were collected and separated within 6 to 8 hours of specimen collection and stored at −80-degree centigrade for four weeks. Batch testing of samples was performed by a senior technician at Muhimbili University of Health and Allied Sciences (MUHAS). Daily calibration of instruments was done following standardized procedures. The MUHAS laboratory participates in the College of American Pathologists proficiency testing programs where three general chemistry panels including lipids and two calibration verification panels are taken annually. Immunologic assessment with CD4+ T-cell count was performed using the FACS Calibur System (Becton Dickinson, San Jose, California, USA).

TB screening was performed to all patients at baseline using a TB screener form containing five questions:

- History of cough for ≥2 weeks
- History of hemoptysis
- History of fever for ≥2 weeks
- Noticeable weight loss for new patients or weight loss ≥ 3.0 kg in a month
- Excessive night sweats for ≥2 weeks.

All patients who were screened positive for TB (had at least one of the abovementioned symptoms) had a chest radiograph and sputum analysis conducted for diagnosis of TB.

### 2.5. Data Collection and Management

Physicians and nurses completed standard forms capturing demographic, clinical, laboratory, and therapeutic information at baseline and follow-up visits. Data reviewers were stationed at each clinic to ensure completeness of data recording by physicians and nurses. Data collected was then entered into a secure computerized database using unique patient identifiers. The database was updated daily by dedicated data entry clerks trained to use a prospective data collection instrument.

Weekly quality assurance checks were performed by the data management team to ensure data accuracy. Data collected for this analysis included baseline demographics, age, weight, height, blood pressure, midupper arm circumference (MUAC), body mass index (BMI), WHO clinical disease stage, and history of prior or current TB.

### 2.6. Outcomes and Definitions

The primary outcome of interest was hypertension, which was defined according to the guidelines for management of hypertension in HIV. The Joint National Committee VII (JNC VII) report released in 2003 categorizes blood pressure as follows: normal blood pressure (systolic (SBP)/diastolic (DBP) < 120/80 mmHg); prehypertension (SBP 120–139 mmHg or DBP 80–89 mmHg); stage 1 hypertension (SBP 140–159 mmHg or DBP 90–99 mmHg); and stage 2 hypertension (SBP ≥ 160 mmHg or DBP ≥ 100 mmHg) [25].

### 2.7. Ethical Approval

The study was approved by institutional review boards for human research at Harvard TH Chan School of Public Health and the Research Ethics Review Committee of the Muhimbili University of Health and Allied Sciences. Patients were recruited for participation and enrolled in the MDH-supported CTCs following written informed consent.

### 2.8. Statistical Analysis

Sociodemographic and cardiometabolic characteristics of the study population were described using mean (SD) or percentage. To examine the factors associated with hypertension, prevalence ratios (relative risks) were estimated using Generalized Estimating Equations (GEE) with the log link function and the binomial variance [26, 27]. All multivariate analyses were adjusted for age (<30, 30–39, 40–49, and ≥50 years); gender (male/female); district (Ilala, Kinondoni, and Temeke); calendar year and season of enrolment; BMI (underweight, normal weight, overweight, and obesity); CD4+ T-cell count (<350, 350–<500, and ≥500); WHO clinical disease stage (I, II, III, and IV); history of TB; and current TB/HIV coinfected. The median score test was used to assess the significance of any trends observed and a Wald test was used for binary variables. The missing indicator method was used in the multivariate models [28]. Statistical analyses were performed with the statistical software package SAS (release 9.2). All tests were two-sided, and a *p* < 0.05 was considered statistically significant.

## 3. Results

### 3.1. Sociodemographic and Clinical Characteristics of the Study Population

A total of 34,111 nonpregnant, HAART naïve patients who had blood pressure measurement taken were included in the analysis. The baseline demographic and clinical characteristics of the study patients are summarized in Table 1. The mean age and BMI of the patients were 36.6 ± 9.5 years and 21.4 ± 4.8 kg/m^2^, respectively, and women constituted two-thirds of the study participants. Three-quarters (75.5%) of the participants had CD4+ T-cell count of less than 350 cells/*μ*L and two-thirds (66.2%) had a WHO clinical disease stage III or stage IV. Nearly quarter of the participants (23.3%) had a positive history of TB and about one-tenth (9.7%) were currently TB/HIV coinfected receiving anti-TB treatment.

### 3.2. Blood Pressure and Prevalence of Hypertension

Results on blood pressure measurements are summarized in Table 2. The mean systolic and diastolic blood pressures were 114.1 ± 18.1 mmHg and 73.1 ± 12.9 mmHg, respectively. More than half (58.2%) of the patients had their blood pressure measurement within the normal range and more than quarter (29.2%) had their blood pressure in the prehypertension range. The prevalence of hypertension (combined stages 1 and 2 hypertension) found in this study population was 12.5%.

### 3.3. Sociodemographic and Clinical Characteristics Associated with Hypertension

The various sociodemographic and clinical factors associated with hypertension among study patients are summarized in Table 3. Older age (≥50 years), male gender, overweight and obesity, CD4+ T-cell count (≥500 cells/*μ*L), and WHO clinical disease stage I were all significantly associated with higher prevalence of hypertension. WHO clinical disease stage IV, history of TB, and being TB/HIV coinfected were significantly associated with a lower prevalence of hypertension.

### 3.4. Relationship between Age, Gender, and Body Mass Index with Prevalence of Hypertension

Results for univariate and multivariate analyses for risk factors associated with hypertension are summarized in Table 4. After adjusting for district (Ilala, Kinondoni, and Temeke), calendar year and season of enrolment, age (<30, 30–39, 40–49, and ≥50 years), gender (male/female), BMI (underweight, normal weight, overweight, and obesity), CD4+ T-cell count (<350, 350–<500, and ≥500), WHO clinical disease stage (I, II, III, and IV), history of TB, and current TB/HIV coinfected, patients aged 40–49 years and those aged ≥50 years had a 43% [ARR 1.43 (95% CI 1.33, 1.53)] and 2-fold [ARR 2.52 (95% CI 1.92, 3.30)] increased risk for hypertension compared to patients aged 30–39 years. Male patients had 10% [ARR 1.10 (95% CI 1.04, 1.17)] increased risk of hypertension compared to female patients. Overweight and obesity were associated with 51% [ARR 1.51 (95% CI 1.40, 1.62)] and 94% [ARR 1.94 (95% CI 1.78, 2.12)], respectively, increased risk for hypertension compared to normal weight patients.

Prevalence of hypertension was significantly lower in patients with immune suppression at baseline. Hypertension was 10% (ARR 0.90; 95% CI 0.83, 0.98) lower in patients with CD4+ T-cell count < 350 cells/*μ*L compared to those with CD4+ T-cell count ≥ 500 cells/*μ*L. Similarly, patients with advanced WHO clinical disease stage had significantly lower risk of hypertension. Patients with WHO clinical disease stages II and III had 12% and 28% decreased risk for hypertension compared to patients with stage I disease. WHO clinical disease stage IV was associated with 42% decreased risk for hypertension compared to stage I disease.

History of TB was observed to be protective against hypertension. Patients with history of TB had statistically significant 14% decreased risk for hypertension compared to patients with no history of TB. On the contrary, patients who were current TB/HIV coinfected had a nonsignificant 5% increased risk for hypertension.

## 4. Discussion

We report an appreciable prevalence of hypertension in a cohort of HAART naïve HIV-infected adults in Tanzania. We found significant associations between older age, male gender, and overweight/obesity with higher prevalence of hypertension. Furthermore, the prevalence of hypertension was inversely associated with level of immune suppression. This study is one among few published studies examining the prevalence of hypertension as one of the key risk factors associated with CVD in HIV-infected population from resource-limited settings.

Arterial hypertension is a major CVD risk factor. However, there are few studies that have analyzed the relationship between blood pressure and HIV infection [7, 8]. In our study, we observed a prevalence of hypertension (combined stages 1 and 2) of 12.5%. The prevalence of hypertension observed in this study is lower than that reported by studies conducted elsewhere in Africa [13, 29–31]. Although we did not compare the prevalence of hypertension to patients on HAART, several studies have reported higher prevalence of hypertension among HIV-infected patients on HAART [29, 30, 32, 33], supporting that HAART is associated with hypertension. Other studies have found no association between HAART use and hypertension [9, 34]. Ogunmola et al. reported no significant difference in the prevalence of hypertension, mean SBP, and mean DBP between HIV-negative, HIV-positive on HAART, and HIV-positive HAART naïve patients [13].

The variability in the prevalence of hypertension observed in our study to that reported by other studies may be explained by several factors, one of them being the cut-off for defining hypertension. For example, in most of these studies, their cut-off point was 160/95 mmHg compared to the definition we used (SBP ≥ 140 mmHg and/or DBP ≥ 90 mmHg). Other factors such as differences in age, ethnicity, and levels of obesity may also explain the variations in the observed prevalence of hypertension. In our study, inclusion of HAART naïve patients only may partly explain the observed low prevalence of hypertension. Further analysis comparing with patients on HAART is warranted.

Several mechanisms have been proposed to explain the link between HIV and CVD [31, 35]. HIV infection, chronic inflammation, hypercoagulability, and platelet activation mediated by HIV infection itself contribute to endothelial dysfunction and subsequent increased CVD risk [15, 36]. It has been proposed that HIV influences endothelial function via activated monocytes and resultant cytokine secretion and via a direct effect of the secreted HIV proteins tat and gp120 [37]. Another mechanism involves free radical physiology, where excess nitric oxide reacts with oxygen radicals to produce peroxynitrite, which then causes oxidative damage to the vascular endothelium, and decreased flow mediated dilation [38]. Use of HAART in patients infected with HIV-1 has been shown to reduce markers of endothelial function and coagulation [15, 31, 35].

We observed a significant association between older age, male gender, and overweight/obesity and increased risk for hypertension. These findings are in agreement with other studies conducted among HIV-infected individuals. In addition to HAART exposure, these studies found male gender, advancing age, high body mass index, greater waist circumference, HIV-hepatitis C coinfection, and ethnicity to be associated with increased risk of developing hypertension and coronary heart disease (CHD) in HIV-infected patients [7, 33]. We have also demonstrated increased mortality among male patients with HIV in one of our publications [39].

We observed an inverse association between advanced HIV disease (defined as low CD4+ T-cell count and advanced WHO clinical disease stage) and risk for hypertension. Participants with low CD4+ T-cell count < 350 cells/*μ*L and those with WHO clinical disease stage IV had lower risk for hypertension. This finding is contrary to results from other studies which show that low CD4+ T-cell count is a risk for CVD [40–43]. The proposed mechanism for association between low immunity and risk of CVD is that chronic inflammation that accompanies uncontrolled or more advanced HIV disease is associated with elevated levels of serum markers of inflammation and increased levels of activated CD4+ T-cells and proinflammatory cytokines that destabilize atherosclerotic plaques leading to CVD event [42, 44].

Participants with history of TB had a 10% decreased risk for hypertension, but this protective effect was not observed among those who were currently TB/HIV coinfected. Few studies have explored the association between TB and CVD risk and have reported contradicting conclusions. An age and sex matched study conducted by Giral et al. [45] showed that past TB was not associated with a higher prevalence of atherosclerotic lesions in patients with hypercholesterolemia patients, while Wu et al. [46] reported that non-CNS TB does not increase the risk of subsequent ischemic stroke. In contrary, Sheu et al. [47] found patients with a diagnosis of TB to be at an increased risk for ischemic stroke. Our finding of history of TB conferring protective effect against hypertension further increases the need for further research to investigate the relationship between TB and CVD and its risk factors.

While the major strength of this analysis is its large sample size, it has several limitations worth mentioning. First, the cross-sectional design of the study does not provide proof of causal association between HIV, immune suppression, and the development of hypertension. Secondly, the analysis was limited to hypertension and obesity. We did not evaluate the contribution of other conversional risk factors such as diabetes, lipid profile, smoking, alcohol drinking, physical activity, dietary, and other lifestyle related factors. Third, inclusion of HAART naïve only in the analysis made it impossible to make comparisons with counterpart HIV-infected patients on HAART or HIV-negative as in other studies [13, 30]. Fourth, selection bias might have been introduced by inclusion of patients who attended MDH-supported CTCs only, thus affecting generalizability of the findings. However, MDH-supported CTCs are publicly accessible and patients attending these clinics may be similar to those attending other public health facilities CTCs in Dar es Salaam. Despite the abovementioned limitations, this study remains an important analysis of blood pressure and its associated risk factors in a large cohort of HIV-infected HAART naïve patients from resource-limited settings.

In conclusion, we observed that older age, male gender, and BMI-defined overweight/obesity were significantly associated with higher prevalence of hypertension even after adjusting for potential confounders. Furthermore, immune suppression (defined by low CD4+ T-cell count and advanced WHO clinical diseased stage) and history of TB were associated with decreased risk for hypertension. Screening and regular monitoring for complications associated with use of HAART among HIV-infected people have been a routine clinical practice. HIV treatment programs are recommended to conduct routine screening and monitoring for hypertension and other CVD risk factors even prior to initiation of HAART especially among healthy-looking individuals. Traditional hypertension control measures such as physical exercise, weight control, and healthy diet should be recommended to HIV-infected individuals. Further research is warranted to investigate the association between TB, immune suppression, and CVD risk observed in this study.

## Figures and Tables

**Table 1:** Sociodemographic and clinical characteristics of the participants.

Characteristic	Mean ± SD or *N* (%)
*Age (years)*	36.6 ± 9.5
*Age categories*	
<30	7760 (22.9)
30–39	14877 (43.9)
40–49	7964 (23.4)
≥50	3317 (9.8)
*Gender*	
Male	11199 (32.8)
Female	22912 (67.2)
*BMI (kg/m* ^*2*^)	21.4 ± 4.8
*BMI-defined categories*	
Underweight (BMI < 18.5 kg/m^2^)	9235 (27.6)
Normal (BMI 18.5–24.9 kg/m^2^)	18160 (54.4)
Overweight (BMI 25.0–29.9 kg/m^2^)	4322 (12.9)
Obesity (BMI ≥ 30 kg/m^2^)	1693 (5.1)
*CD4+ cell count (cells/µL)*	
<350	23050 (75.5)
350–<500	3748 (12.3)
≥500	3720 (12.2)
*WHO clinical disease stage*	
Stage I	4910 (14.9)
Stage II	6228 (18.9)
Stage III	15128 (45.9)
Stage IV	6686 (20.3)
*History of TB infection*	
Yes	7739 (23.3)
No	25539 (76.7)
*Current TB/HIV coinfection*	
Yes	3245 (9.7)
No	30246 (90.3)

BMI: body mass index; BP: blood pressure; CD4+: cluster of differentiation 4; HIV: human immunodeficiency virus; TB: tuberculosis; WHO: World Health Organization.

**Table 2:** Mean systolic and diastolic blood pressures and prevalence of hypertension.

Variable	Mean ± SD or *N* (%)
*Systolic blood pressure, SBP (mmHg)*	114.1 ± 18.1
*Diastolic blood pressure, DBP (mmHg)*	73.1 ± 12.9
*Blood pressure categories*	
Normal BP	19859 (58.2)
Prehypertension	9967 (29.2)
Stage 1 hypertension	2668 (7.8)
Stage 2 hypertension	1617 (4.7)
*Hypertension*	
Yes	4285 (12.5)
No	29826 (87.4)

BP: blood pressure; SD: standard deviation.

**Table 3:** Sociodemographic and clinical characteristics associated with hypertension.

Parameter	All *N* = 34,111	Normotensive *N* (%)	Hypertensive *N* (%)	*p* value
*Age (years)*				**<0.001**
<30	7760	7131 (91.9%)	629 (8.1%)	
30–39	14877	3289 (89.3%)	1588 (10.7%)	
40–49	7964	6756 (84.8%)	1208 (15.2%)	
≥50	3317	2505 (75.5%)	813 (24.5%)	
*Gender*				**<0.001**
Male	11199	8423 (75.2%)	2776 (24.8%)	
Female	22912	21409 (93.4%)	1503 (6.6%)	
*BMI-defined obesity*				**<0.001**
Underweight (BMI < 18.5 kg/m^2^)	9235	8643 (93.6%)	592 (6.4%)	
Normal (BMI 18.5–24.9 kg/m^2^)	18160	15884 (87.5%)	2276 (12.5%)	
Overweight (BMI 25.0–29.9 kg/m^2^)	4322	3440 (79.6%)	882 (20.4%)	
Obesity (BMI ≥ 30 kg/m^2^)	1693	1228 (72.5%)	465 (27.5%)	
*CD4+ cell count (cells/µL)*				**<0.001**
<350	23050	20414 (88.6%)	2636 (11.4%)	
350–<500	3748	3177 (84.8%)	571 (15.2%)	
≥500	3720	3120 (83.9%)	600 (16.1%)	
*WHO clinical disease stage*				**<0.001**
Stage I	4910	3982 (81.1%)	928 (18.9%)	
Stage II	6228	5234 (84.0%)	994 (16.0%)	
Stage III	15128	13392 (88.5%)	1736 (11.5%)	
Stage IV	6686	6139 (91.8%)	547 (8.2%)	
*History of TB*				**<0.001**
Yes	7739	6993 (90.4%)	746 (9.6%)	
No	25539	22097 (86.5%)	3442 (13.5%)	
*Current TB/HIV coinfection*				**<0.001**
Yes	3245	2940 (90.6%)	305 (9.4%)	
No	30246	26345 (87.1%)	3901 (12.9%)	

BMI: body mass index; CD4+: cluster of differentiation 4; HIV: human immunodeficiency virus; TB: tuberculosis; WHO: World Health Organization.

**Table 4:** Univariate and multivariate adjusted demographic, body mass index, and clinical and immunological factors associated with prevalence of hypertension.

	Unadjusted RR (95% CI)	*p* value	Adjusted RR (95% CI)	*p* value
*Age group (years)*		**0.002**		**<0.001**
15–29	0.76 (0.70–0.83)		0.74 (0.67–0.81)	
30–39	Reference		Reference	
40–49	1.42 (1.32–1.52)		1.43 (1.33–1.53)	
50	2.30 (2.14–2.49)		2.52 (1.92–3.30)	
*Gender*		**<0.001**		**0.002**
Female	Reference		Reference	
Male	1.11 (1.05–1.18)		1.10 (1.04–1.17)	
*BMI-defined obesity*		**0.003**		**<0.001**
Underweight (BMI < 18.5 kg/m^2^)	0.51 (0.47–0.56)		0.57 (0.52–0.62)	
Normal (BMI 18.5–24.9 kg/m^2^)	Reference		Reference	
Overweight (BMI 25.0–29.9 kg/m^2^)	1.63 (1.52–1.74)		1.51 (1.40–1.62)	
Obesity (BMI ≥ 30 kg/m^2^)	2.19 (2.00–2.38)		1.94 (1.78–2.12)	
*CD4+ cell count (cells/μL) *		**<0.001**		**0.003**
<350	0.49 (0.44–0.54)			
350–<500	0.77 (0.72–0.82)			
>500	Reference		Reference	
*WHO Clinical disease stage*		**<0.001**		**<0.001**
Stage I	Reference		Reference	
Stage II	0.83 (0.77–0.90)		0.88 (0.81–0.96)	
Stage III	0.58 (0.54–0.63)		0.72 (0.66–0.78)	
Stage IV	0.42 (0.38–0.47)		0.58 (0.52–0.64)	
*History of TB*		**<0.001**		**<0.001**
Yes	0.72 (0.66–0.77)		0.86 (0.78–0.94)	
No	Reference		Reference	
*Current TB/HIV coinfection*		**0.410**		**0.420**
Yes	1.08 (0.89–1.18)		1.05 (0.92–1.20)	
No	Reference		Reference	

BMI: body mass index; CI: confidence interval; HIV: human immune deficiency virus; RR: risk ratio; TB: tuberculosis; WHO: World Health Organization.

The final model for multivariate analyses included district (Ilala, Kinondoni, and Temeke), calendar year and season of enrolment, age (<30, 30–39, 40–49, and ≥50 years), gender (male/female), BMI (underweight, normal weight, overweight, and obesity), CD4+ cell count (<350, 350–<500, and ≥500), WHO clinical disease stage (I, II, III, and IV), history of TB, and current TB/HIV coinfected. The median score test was used to assess the significance of any trends observed and a Wald test was used for binary variables.
